# Levels of Anti-HBs Antibody in HBV-Vaccinated Students Enrolled in the Faculty of Medicine, Dentistry and Health Professions of a Large Italian University

**DOI:** 10.1155/2015/712020

**Published:** 2015-01-05

**Authors:** Sabina Sernia, Marina Ortis, Tranquillo Antoniozzi, Emanuele Maffongelli, Giuseppe La Torre

**Affiliations:** ^1^Center of Occupational Medicine, Sapienza University of Rome, 5-00185 Rome, Italy; ^2^Department of Public Health and Infectious Diseases, Sapienza University of Rome, 5-00185 Rome, Italy; ^3^Eleonora Lorillard Spencer-Cenci Foundation, 00185 Rome, Italy

## Abstract

*Background*. Prophylaxis against hepatitis B virus (HBV) addressed to students of the faculties of health professions has received great attention. * Objectives*. The present study aims to assess vaccination coverage against hepatitis B in healthcare professionals in training. * Materials and Methods*. A retrospective study was carried out using data from the students of medicine, dentistry, and health professions. * Results*. 4180 vaccination certifications were examined through the internal database. Significant differences (<0.0001) emerge between the number of doses applied and the antibody level. 50.4% of the students have nonprotective antibody levels (<10 IU). The age of the first dose significantly influences the level of coverage, resulting in more coverage in those vaccinated with earlier onset (1–10 years). Antibody levels are not significantly different by type of course; the levels of noncoverage are present in 44.4% of the students of medicine and dentistry and in 50.6% among those belonging to the health professions. * Conclusions*. This study represents one of the first experiences in Italy on vaccination against HBV and the relationship between doses of vaccination and antibody titer in the biomedical students that can configure a step forward in the real-time monitoring in order to establish a register of vaccination.

## 1. Background

Prophylaxis against hepatitis B virus (HBV) addressed to the students of the Faculty of Medicine and Health Professions has received great attention in Italy. The law 165/1991 established the mandatory nature of this type of prophylaxis for all newborns in the first year of life and for the twelve-year olds and the mandatory vaccination for all subjects during the twelfth year of age; the specific mandatory vaccination of adolescents ended in 2003 but continues to be recommended and offered to health workers and others [1].

HBV infection shows a high frequency in some population groups, including homeless and immigrants, and the WHO foresees them to remain in 2030 in the third and fourth places among the causes of death from infectious diseases in industrialized countries [1].

Health care workers, because of their contact with patients and with potentially infectious materials, have risk of exposure to infectious diseases, some of which are preventable by vaccination. The goal of immunization for appropriate intervention in the health workforce is critical to prevent and control infections. Vaccination programs well set up may substantially reduce the number of sensitive operators and the resulting risks to acquire occupational infections and to transmit them to patients or other health professionals. HBV represents the infection due to which the occupational hazard for health care is maximum/highest, and it is essential to provide vaccination to all exposed individuals, who receive 3 doses of vaccine at 0, 1, and 6–12 months, possibly before commencing operations at risk. If there is an urgent need to be exposed to infection risk, it is possible to be vaccinated with a fast 4-dose schedule (0, 1, 2, and 12 months), which guarantees a high probability of protective response already after the first 3 doses. There is also the need to verify that, to have certainty of establishment of immunological memory, the seroconversion (presence of anti-HBs) has been accomplished/done one month after the last dose. For health students and health care professionals born after 1980, who are supposed to have been vaccinated against hepatitis B at the age of twelve, the implementation/accomplishment of the test is recommended to check HBsAb level before commencing operations at risk. A positive result testifies the presence of immunological memory and does not require further action. In contrast, in those with negative test, a single dose of vaccine and a new antibody control after one month are recommended. Antibodies-HBs indicate presence of immunological memory; their persistent negativity indicates the need to complete the vaccination series with two additional doses followed by a new serological testing after one month. “No responder” subjects after a vaccine series may be administered up to 3 additional doses (at 0, 1, and 6 months) to attempt conferring protection. Recently, for these subjects, a new vaccination schedule has been proposed, which involves the administration of 2 doses simultaneously in deltoid muscles, followed by a similar dose after 2 months, and serological testing to verify eventual seroconversion (anti-HBs ≥ 10 mIU/mL) at a distance of two additional months. Vaccine for HBV is mandatory for physicians, nurses, and other paramedical staff in five countries and is recommended in all other countries; it was mandatory for medical and nursing students in five countries and is recommended in another nine. Serum prevaccination testing is performed in six countries. Vaccination program most often used was 0, 1, and 6 months. Combined vaccine (hepatitis A virus/HBV) was used in 10 countries. Postvaccination serum test was performed in 14 countries. Data on vaccination coverage for HBV were available in 11 countries and were published in five of them. The coverage was 85 to 93%. These results show legislative variables of the EU and their subsequent implementation in European countries. Greater consultation among member countries of EU could help to improve the way in which this problem is treated. A set of measures and interventions that include the introduction of immunization programs against HBV infection and increased coverage of immunization in health care workers can help to further reduce the transmission of HBV in health care workers [2].

There is sufficient evidence that the levels of anti-HBs antibody are related to the number of doses of vaccine administered [3, 4], but the studies conducted in Europe are few and old [5, 6]. So, the aim of this work was to evaluate the levels of anti-HBs antibody in HBV-vaccinated students and the associated factors in biomedical faculties at “Sapienza” University of Rome.

## 2. Materials and Methods

**Figure 1:**
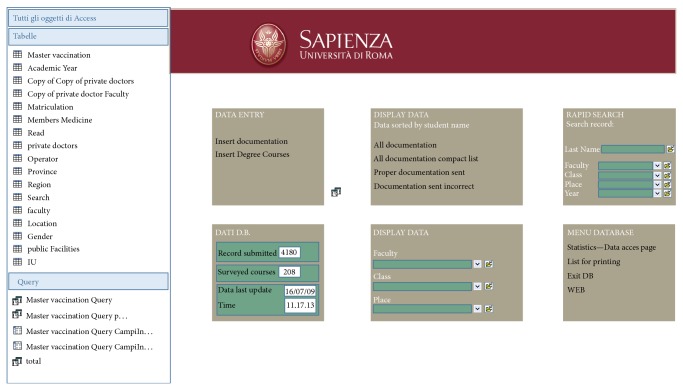
Access Database of the Center for Occupational Medicine.

**Figure 2:**
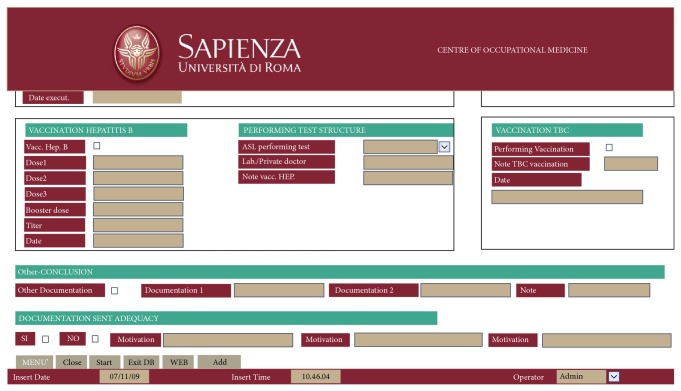
Access Database Center of Occupational Medicine, with the specifications on the HBV vaccination.

A retrospective epidemiological study was carried out, using data of the students of Medicine, Dentistry and Health Professions of Sapienza University of Rome. These data were extracted from a special database in Access created by the Centre of Occupational Medicine (CMO) of the university (Figure 1) and in particular from the table relating to vaccination against hepatitis B (Figure 2). The CMO was established in 2000 with the specific aim of working in the field of prevention and health surveillance of the university personnel, including the students, and the electronic database was established in 2009.

The data-entry process in this database was a gradual process over the years, collected by documentation required to the biomedical students sent/delivered to the Centre of Occupational Medicine, which functioned as a collection “basket” and as processing point of the above documents. According to enrollment announcement established at Sapienza University of Rome, the biomedical students need to present documentation concerning the presence of hepatitis B (vaccination and titers of anti-HBs antibody) and tuberculosis (vaccination and Mantoux skin test) prophylaxis. As far as hepatitis B prophylaxis is concerned, the effectiveness of the vaccination was assessed by comparing anti-HBs antibody levels in students for whom the number of vaccine doses (1, 2, 3, and 4 doses) was reported.

The anti-HBs antibody levels were detected using the anti-HBs ELISA test (enzyme immunoassay diagnostic kit for in vitro qualitative detection of antibody to hepatitis B surface antigen in human serum or plasma).

Inadequate immune response to HBV vaccination was considered if quantitative anti-HBs level was <10.0 mIU/mL, according to the CDC [7].

Since the titer was not normally distributed, we categorized this variable into three groups according to the following cut-off points: 10 mIU/mL and 1121.63 mIU/mL (median of the titers). So, differences by level of antibody titer were analyzed by the chi-square test. The Yates correction was used if the expected cell frequency was below 5. Finally, a multivariate logistic regression analysis was used in order to verify the association of independent variables with the seroconversion (anti-HBs antibody levels >10 mIU/mL). Results are presented as odd ratio (OR) and 95% confidence intervals (95% CI).

Level of significance was set at *P* < 0.05. Statistical analysis was carried out through the package/program SPSS for Windows.

The study received the approval of the local ethical committee.

## 3. Results

A total of 4180 vaccination certifications in the academic years between 2003/2004 and 2008/2009 were examined through the internal database of Sapienza. Of 4150, 85% of the forms/cards were complete, while 15% were incomplete. A total of 3527 cards/forms/data were analyzed, and 2670 (75.7%) showed the suitability of the documentation submitted, as far as the combination between age, gender, and number of doses is concerned. Overall, 97.3% of students presented certification relating to vaccination against hepatitis B. The sample in question consisted mainly of female subjects with a mean age of 25.4 years. The bachelor of origin was represented mainly by Nursing, Medicine and Physiotherapy. Considering the number of doses, 3479 (98.6%) students resulted as vaccinated with 1 dose, 3252 (92.2%) with 2 doses, 2920 (82.8%) with 3 doses, and 193 (5.5%) with 4 doses. Data collected show extreme variability in procedures and methods of compilation of this form of certification; in 3312 cases the age of the first dose was reported (median age 11.68 years); anti-HBs antibody titer was present in 759 individuals. However for the association between number of doses and titer level only in 369 students was the combination of these data available, since in 369 students the number of vaccine doses practiced was specified. Significant differences (*P* < 0.0001) arise between number of doses applied and antibody level (Table 1). 50.4% of students have nonprotective antibody levels (<10 IU/L), while optimal levels of protection are achieved by those who carried 3 or 4 doses of vaccine, with protection rates, respectively, 54 and 57%. Level of vaccine coverage shows no differences by gender (*P* = 0.998) (Table 1), while it increases particularly in last academic years (*P* < 0.001) (Table 1). 49.1% of males and 49.4% of females are protected (Table 1). It is interesting to note that the level of coverage is significantly influenced by the age at first dose; those vaccinated with earlier onset (1–10 years) have higher coverage (68.8% compared to 47% in individuals vaccinated from the age of 11), even if this figure, cross-checking the two variables, is present for 279 students (*P* = 0.003) (Table 1). Table 1 shows the relationship between age at the time of enrolment and antibody level, which was observed in 348 individuals. While overall lower level of coverage does not reach 50.3%, in the age group of 21–24 years at enrolment, that level drops significantly to 37.1% (*P* = 0.010). Lastly, it is necessary to consider that antibody levels are not significantly different by type of course of study: levels of a shortfall are present in 44.4% of the students of Medicine and Dentistry and 50.6% among those belonging to Health Professions (*P* = 0.763). The multivariate logistic regression analysis revealed that variables significantly associated with seroconversion (>10 mIU/mL) were the number of doses (AOR = 3.91; 95% CI: (1.44–10.57) for at least 3 doses), the younger age group (AOR = 2.44; 95% CI: 1.41–4.35, for 1–10 years old), and the more recent academic year (AOR = 17.0; 95% CI: 8.23–35.2, for academic year since 2007) (Table 1).

## 4. Discussion

In Italy, the rule of law on vaccination in health care workers is regulated by Legislative Decree 9, April 2008, number 81: “The employer, upon advice of the occupational physician, takes special protective measures for those workers who, as well as for personal health reasons, require special protection measures, including the provision of effective vaccines for those workers who are not already immune to the biological agent present in the process, to be administered by the competent physician, the occupational physician is therefore responsible for identifying and carrying out the vaccination for health care personnel. In other cases (e.g., flu vaccination) active immunization plays an important role, not only as protection of the individual operator, but above all as guarantee to patients as well, to which the operator could transmit any infections, causing serious damage and even fatal cases. In view of all this, to all health care professionals and students who attend degree courses and diploma in health area vaccination against HBV is strongly recommended. Currently the National Prevention Plan (Piano Nazionale della Prevenzione, PNP) recommends the implementation of HBV vaccination for health care personnel to newly recruited staff in the National Health Service and employed in National Health Service already engaged in activities with higher risk of infection and in particular employed in departments of haemodialysis, intensive care, oncology, surgery, obstetrics and gynaecology, infectious diseases, haematology, analysis laboratories, blood centres, operating rooms, dental offices, medical examiner and autopsy rooms, first aid, health care in prisons, trainees, work, students, and volunteer work in the health sector.

A study recently published by La Torre er al. [1] conducts comparison of admission notices of academic years between 2003/2004 and 2008/2009, through consultation/examination of the institutional website of the Ministry of Education, University and Research (http://www.istruzione.it/), selecting then the area dedicated to Sapienza University. Using this method it was possible to download the admission notices to degree courses in Medicine, Dentistry and Health Professions. In the admission notices evaluated in this study references relating to hepatitis B vaccinations were carefully searched to get an overview of initiatives in favour of the student population against these infectious diseases. The main strength of this study is that for the first time the association between doses of HBV vaccination and anti-HBsAg titers was carried out in biomedical students in Italy. The present study represents a significant step in the evaluation of vaccination coverage against hepatitis B for a significant proportion of health professionals in training. Important critical issues arise which should be carefully underlined:

1. inconsistency of the record is high, at about one-sixth of the total;
2. vaccination status is not optimal, with 17% of students not adequately vaccinated;
3. protective antibody level is known only in 10.7% of cases:
  - not being specified in the obligations vaccine,
  - being expensive.

In perspective, it is useful, at least for biomedical students, to envisage a revision of the notice of registration, covering specific guidance on documentation of vaccination, including the determination of antibody titers.

Our study is not in agreement with a similar study conducted in Italy by Chiara et al. [8] whose rate of production of protective antibodies against HBV is inversely correlated with age at vaccination. In contrast, our study confirms results from other international organizations. The national program of vaccination for the virus (HBV) was launched in Taiwan in 1984. Some authors point out that only a small number of vaccinated people have lost their immune memory after 16 years, suggesting that some students may benefit from vaccination before proceeding to clinical practice [7]. Moreover, it was found that persistence of immunological memory independent of antibody titer is evident after a vaccination dose practiced in those who possess a titer anti-HBsAg <10 IU/L from 10 to 15 years after vaccination [9]. A similar study was at the University of Monza analyzing university students of Health Professions, evaluating antibody titer and possibly administering a vaccination if it was found to be below 10 mIU/mL [10]. Every year in the period between 2000 and 2006, incoming students of the five-year nursing program at the University of Fooyin were tested for their state of HBsAg and anti-HBs using microparticles of a commercially available immunoassay kit. The seroprevalence of HBsAg(+) showed a significant downward trend, dropping by 57% from 4.9% in 2000 to 2.1% in 2006. However, the seroprevalence of anti-HBs(+) showed a significant downward trend, dropping by 49% from 77.1% in 2000 to only 39.7% in 2006. With the relatively low seroprevalence of anti-HBs(+) of future health professionals and the high endemicity of HBV in Taiwan, the recommendation of the serology tests in students before they move ahead in their clinical practice is prudent [11]. Similarly, the results of a study aimed at evaluating the impact of the vaccination program established in Malaysia since 1989 in response to the needs feared by the WHO to reduce the seroprevalence of HBsAg and HBV-related chronic hepatitis were recently published. In the period 2005–2011, 2923 students belonging to the faculties of Medicine, Dentistry and Health Professions have been analyzed, measuring serum levels of HBsAg, HBsAb, HBcAb, and IgG. During the test period, prevalence of HBsAg had a steady decrease from 1.27% in 2005 to 0 in the years 2010 to 2011 in line with the increased proportion of students vaccinated following the establishment of the prevention program [12].

As far as possible limitations of this study are concerned, we have to admit that the lack of data concerning the titer of anti-HBsA is an issue, and the results could be not representative of all the biomedical students in our university. However, the results of this research let the Sapienza University be able to modify the enrollment announcement for the academic year 2013-2014 and in the future we are sure these data will be more. Moreover, another critical point was lack of data collection process, and we used in the academic year 2013-2014 an electronic form for this purpose that the biomedical students needed to fill in mandatorily.

## 5. Conclusions

Our study represents one of the first experiences in Italy on vaccination against HBV and correlation between doses of vaccination and antibody titer in the biomedical students. This investigation shows numerous shortcomings in past public health policy associated with HBV vaccination; however this experience allowed Sapienza University to better face with this issue and to represent an example for other universities in Italy. We believe that this experience could be a major step forward in monitoring vaccination status integrated with the institution of a register of vaccinations. The authors hope that it will allow an appropriate and unique archiving of the population, a real-time monitoring of the vaccination status vaccination, and the linkage/connection with the local health authorities.

## Figures and Tables

**Table 1:** Univariate and multivariate analysis of level of anti-HBsAg antibodies according to sociodemographic factors, number of doses of HBV vaccination, academic year, and type of biomedical students.

	Titers (mIU/mL) (%)			*P*	Crude OR (95% CI)	Adjusted OR (95% CI)
	0–9	10–1121.63	>1121.64			
Gender						
Males	54 (50.9)	46 (43.4)	6 (5.7)	0.998	1	1
Females	132 (50.6)	114 (43.7)	15 (5.7)		1.01 (0.65–1.59)	1.11 (0.69–1.78)
Age group dose 1						
1–10 years	24 (31.2)	46 (59.7)	7 (9.1)	0.003	**2.70 (1.51**–**4.76)**	**2.44 (1.41**–**4.35)**
11–17 years	107 (53.0)	87 (43.1)	8 (4.0)		**1**	**1**
Age group						
≤20	88 (56.4)	59 (37.8)	9 (5.8)	0.010	1^**^	1^**^
21–24	39 (37.1)	61 (58.1)	5 (4.8)			
≥25	48 (55.2)	32 (36.8)	7 (8.0)		0.77 (0.47–1.25)	1.17 (0.67–2.04)
Number of doses						
1	22 (78.6)	5 (17.9)	1 (3.6)	0.001^*^	1	1
2	20 (71.4)	8 (28.6)	0 (0.0)		1.47 (0.43–4.96)	1.4 (0.40–4.86)
3	129 (46.4)	134 (48.2)	15 (5.4)		4.30^∧^ ** (1.7 – 10.91)**	3.91^∧^ ** (1.44–10.57)**
4	15 (42.9)	15 (42.9)	5 (14.3)			
Academic year						
2003-2004	2 (100.0)	0 (0.0)	0 (0.0)	0.001^*^		
2004-2005	2 (66.7)	1 (33.3)	0 (0.0)			
2005-2006	30 (78.9)	8 (21.1)	0 (0.0)			
2006-2007	140 (61.9)	78 (34.5)	8 (3.5)		1	1
2007-2008	6 (16.7)	26 (72.2)	4 (11.1)		**17.69**°** (8.5**–**36.97)**	**17.0**°** (8.23**–**35.2)**
2008-2009	2 (10.6)	14 (73.7)	3 (15.7)			
2009-2010	4 (8.9)	35 (77.8)	6 (13.3)			
Type of students						
Health Professions	182 (50.6)	158 (43.9)	20 (5.6)	0.763	1	1
Medicine	4 (44.4)	4 (44.4)	1 (11.2)		0.93 (0.47–1.86)	0.85 (0.46–1.89)

^*^Yates correction; ^**^the reference group is age ≤24 years; ^∧^the OR is related to 3-4 doses versus 1 dose; °the OR is related to 2007 and over versus before 2007.

## Implication for Health Policy/Practice/Research/Medical Education

Studying the relationship between doses of vaccination and antibody titer in the biomedical students would help to configure a step forward in the real-time monitoring, in order to define the status by vaccination, and allow the establishment of a register of vaccination and linkage to local health facilities.
